# High prevalence of blood donor test-seeking behavior among health sciences undergraduate students

**DOI:** 10.1590/0037-8682-0476-2019

**Published:** 2020-04-22

**Authors:** Miriane Lucindo Zucoloto, Edson Zangiacomi Martinez

**Affiliations:** 1 Universidade de São Paulo, Faculdade de Medicina de Ribeirão Preto, Departamento de Medicina Social, Ribeirão Preto, SP, Brasil.

**Keywords:** Blood donation, Test-seeking behavior, HIV, Students

## Abstract

**INTRODUCTION:**

This study investigated the prevalence of blood donors’ test-seeking behavior and related factors among health sciences undergraduate students.

**METHODS:**

A total of 750 students were invited. Data regarding sociodemographic and behavioral characteristics, blood donation practices, and test-seeking behavior were collected.

**RESULTS:**

Of the invited students, 341 (45.5%) agreed to participate and answered questions regarding test-seeking behavior. The sample comprised 83.1% females, 96.8% singles, 87.2% heterosexuals, and 32.6% of them had previously donated blood. A high prevalence of blood donor test-seeking behavior (14.4%; 95% CI: 10.8%-18.5%) was observed and associated with blood donation practices.

**CONCLUSIONS:**

Test-seeking behavior was common among the interviewed students, thereby highlighting the importance of developing a better understanding of its determinants to prevent this behavior in key populations.

Despite the evolution of serological tests in recent years and new technologies that have been utilized for the early diagnosis of infected individuals^1^, the risk of viral transmission by blood transfusions still persists. Strategies to ensure transfusion safety include the screening of donors in an attempt to prevent donations from those who are likely to be in the window stage of infectious diseases. However, despite these efforts, hemotherapy services are often sought for the diagnosis of communicable diseases by people at risk, thereby greatly increasing the transfusion risk to blood component recipients^2^. A lack of knowledge about immunological window periods, sites that are suitable for serological tests, and difficulties when reporting oft-stigmatized or illegal behaviors associated with exposure to risk factors are posited as the main reasons for the test-seeking behavior of blood donors. Previous studies in different populations have identified a prevalence of 1%-9% of test seekers among blood donors^3^ ^,^ ^4^. In Brazil, the prevalence of test seekers found among donors to the Pró-Sangue Foundation of São Paulo was 9%. It was revealed as a more common behavior among men, donors with low income, and those with less than 8 years of schooling^4^. In addition, among Brazilian donors who were carriers of infectious diseases, 14%-54% admitted that they were motivated to donate blood in order to receive serological tests^5^. 

Alarming data about attempts to receive serological testing in blood banks at the suggestion of healthcare professionals have been reported^6^. In a recent study conducted in four large Brazilians blood banks, donors who had tested HIV positive and those previously refused for donation represented 12.6% and 11.8% of all cases, respectively. Evidence showed that they had sought blood bank testing after suggestions from a physician (69.1% and 61.5% of the cases, respectively), followed by “someone from the health department” (21.4% and 18.4%, respectively)^6^.

The few previous studies that focused on test-seeking behavior were conducted among the blood donor population. Therefore, there is a significant gap in the scientific literature about the investigation of this behavior outside of blood banks. In addition, some populations of specific interest in the HIV transmission cycle have never been investigated in this context. 

It is known that the HIV epidemic has spread considerably among young Brazilians. In the period 2007-2018, the majority of observed cases of HIV detection (52.6%) were in the 20-34 year age range. In addition, it is estimated that a substantial proportion of young adults infected in Brazil are unaware that they are living with the virus^7^. They have therefore been classified as a group particularly vulnerable to sexually transmitted infections (STIs) considering the biopsychosocial and programmatic aspects and the consequences of several changes that young people undergo during their growth process. Additionally, the university period is a phase of expressive change that foments the emergence and consolidation of certain risk behaviors, such as alcohol consumption, drug usage, and casual sexual encounters, the latter being especially worrisome when combined with the low use of contraceptives and high numbers of partners reported in some studies^8^ ^-^ ^10^. 

Based on the epidemiological and demographic profile of new HIV infections in Brazil, the alarming data regarding the test-seeking behavior, and the relevance of the health sciences undergraduate student population in the discussion of motivational factors for test-seeking, the present study investigated the blood donor test-seeking behavior among students at Ribeirão Preto Medical School.

To achieve this objective, a cross-sectional study was conducted with a sample of undergraduate health sciences students from the University of São Paulo, Ribeirão Preto campus, a public university. A total of 750 undergraduate students enrolled in occupational therapy, speech therapy, nutrition, and physiotherapy courses were invited to participate. 

The questionnaire was self-administered by students in their classrooms at previously agreed times with their teachers. Completion time was approximately fifteen minutes. The variables were as follows: the sex registered at birth, sexual orientation, age, type of housing, religion, religious affiliation, self-perception of religiosity, marital status, self-perception of health, previous blood donation history, and smoking status. Blood donor test-seeking behavior was recorded through one question: “Have you ever sought to donate blood with the sole purpose of receiving test results for some disease?” (yes or no). Only students who answered this question (either yes or no) were included in the study. 

The study protocol was approved by the local ethics committee. The prevalence of test-seeking behavior was estimated by confidence intervals (95% CI). The association of test-seeking behavior and the variables of interest were assessed through Fisher’s exact test. The level of significance adopted was 5%. 

Of the 750 students invited, 341 (45.5%) agreed to participate and answered the question about test-seeking behavior. The sample comprised predominantly students aged 18-25 years who were mostly female (83.1%), single (96.8%), and heterosexual (87.2%). Of those, 45.3% lived in student residences (dormitories), 78.3% classified their health as good, 71.4% reported having a religion (Catholic was the most prevalent), and 49.8% declared themselves as moderately religious persons. Regarding their blood donation practices, 32.6% had donated blood at some point in their lifetime. The search for tests at centers offering hemotherapy services was higher for those who had never donated blood (20.1%) followed by those who declared themselves unable to donate blood (18.8%).

Regarding test-seeking behavior, 49 (14.4%) students declared that they had already sought places to donate blood for the primary purpose of receiving serological tests (95% CI: 10.8%-18.5%). The analyses of the association of test-seeking behavior and the variables of interest are presented in Table 1. 

**TABLE 1:** Characterization of the sample of health sciences undergraduate students (n = 341) and the association of test-seeking behavior with sociodemographic and behavioral variables. Ribeirão Preto, São Paulo, 2018.

		Test seekers		
	Total	n	%	p-value
**Course**				
Speech Therapy	84	16	19.0	0.50
Nutrition	75	8	10.7	
Physiotherapy	141	19	13.5	
Occupational Therapy	41	6	14.6	
**Age group (years)**				
18-20	165	28	17.0	0.21
21-25	153	17	11.1	
25+	15	3	20.0	
**Sex registered at birth**				
Male	57	13	22.8	0.06
Female	280	36	12.9	
**Sexual orientation**				
Heterosexual	286	41	14.3	0.05
Male homosexual	12	2	16.6	
Female homosexual	6	2	33.3	
Male bisexual	2	1	50.0	
Female bisexual	22	0	0.0	
**Residence**				
In a dormitory (student residence)	154	23	14.9	0.33
With relatives or family of origin	88	15	17.0	
With a partner (husband/wife/girlfriend/boyfriend)	7	1	14.3	
In a pension or boarding school	15	4	26.7	
With other family members (relatives)	10	1	10.0	
Alone	66	5	7.6	
**Religion**				
Yes	242	36	14.9	0.74
No, but believes in God	49	10	20.4	
No, and does not believe in God	24	0	0.0	
Agnostic	24	3	12.5	
**Which religion? (*n* = 242)**				
Catholic	144	22	15.3	0.70
Evangelical	44	9	20.5	
Protestant	5	0	0.0	
Spiritist	44	5	11.4	
Other	5	0	0.0	
**Self-perception of religiosity**				
Very religious	25	3	12.0	0.19
Moderately religious	168	28	16.7	
Somewhat religious	73	13	17.8	
Not religious	71	5	7.0	
**Marital status**				
Single	335	49	14.6	0.60
Widowed / living together	6	0	0.0	
**Self-perception of health**				
Good	263	38	14.4	0.85
Regular	73	11	15.1	
**Blood donation practice**				
Has already donated blood	110	4	3.6	<0.01
Has never donated blood	179	36	20.1	
Declared as unable to donate	48	9	18.8	
**Smoking status**				
Smoker	31	3	9.7	0.47
Has never smoked	274	39	14.2	
Has smoked previously	29	6	20.7	

The prevalence of test-seeking behavior found in the present study is similar to that found in the HIV positive donor population^11^ and is considered high compared with previous studies of test-seeking behavior among blood donors^2^ ^,^ ^4^ ^,^ ^12^. In addition, this behavior was more frequent among students who had never donated blood, thus indicating that although test-seeking behavior was manifested, blood banks in these cases were able to detect potential test candidates, exclude donations after careful screening, and increase transfusion safety. However, 3.6% of those who reported giving previous donations confessed to being motivated by a search for tests, thus demonstrating that blood banks should be aware of this behavior. According to previous related studies^5^ ^,^ ^13^, the circumstances were different and considered a relevant concern for blood service centers. For example, in studies performed among blood donors in Fundação Pró-Sangue in São Paulo, high risks of contracting sexually transmitted infections were detected among the group of test seekers approved to donate blood that were not detected and deferred by current screening questions^5^ ^,^ ^13^. 

Regarding the demographic profile of test seekers identified in previous studies among blood donors in Brazil, there is a consensus that men with low income and less than eight years of schooling are more likely to seek tests in blood banks. However, results from our sample of health science undergraduate students did not reveal evidence of associations between this behavior and the “sex” or “social class” variables. Nevertheless, the demographic profile of the sample may have influenced the results because of the minimal participation of male students (approximately 17%), an inherent characteristic of the types of courses included in the study. 

It is expected that undergraduate students, especially those attempting health sciences courses, have substantial knowledge about infectious diseases, transmission, and prevention methods. They can potentially play an important role in the diffusion of knowledge regarding health promotion; however, lower than expected levels of knowledge for these topics have been reported in recent studies^14^. As mentioned, it is also common to find studies reporting elevated risk behaviors related to STIs of these students, including low frequency of condom use, high numbers of partners, sexual intercourse under the influence of alcohol/drugs, and sexual intercourse with barely or only recently known persons^8^ ^,^ ^9^. 

These findings, when added to the narrative of healthcare professionals account for a significant proportion of persons seeking tests at blood banks^6^, signal a need for further studies about this topic at earlier stages in the university environment and in the context of healthcare systems. Understanding the determinants of test-seeking behavior is paramount for the maintenance of transfusion safety in Brazil.

Ribeirão Preto does have several facilities for voluntary counseling and testing (VCTs) where the population can be tested for infectious diseases free of charge; however, studies suggest that many believe that HIV testing and the receipt of results is faster at blood centers. Also, many persons may wish to avoid the disclosure of stigmatized risk behaviors, loss of privacy, and subsequent risk reduction counseling that accompanies the public system^13^ ^,^ ^15^. In addition, according to Gonçalez et al.^5^, blood centers are seen as more socially acceptable places for testing by some individuals because they are not associated with any stigma, and persons donating blood receive positive feedback because they are helping society.

Our data were collected from a blood bank located on a university campus that could conceivably make the search for tests more convenient for students. This may be considered a limitation of the study. In addition, the lack of information about the reasons for test-seeking behavior, reasons for refusal of blood donors, or the factors leading to ineligibility seem to negate further discussions about the determinants of this behavior in the sample. However, considering these initial results, we can already hypothesize that blood donors’ test-seeking behavior may be common among all undergraduate students regardless of whether they are enrolled in health sciences courses. Our findings highlight the importance of conducting further studies in order to better understand the determinants of test-seeking behavior with the ultimate goal of improved prevention. In addition, the present study confirms the need of educational programs regarding the blood donation process for young people as well as the importance of disseminating information about access to serological tests and suitable testing locations, such as a center of voluntary counseling and testing (VCT).
